# Barriers to Efficient Foliar Uptake of dsRNA and Molecular Barriers to dsRNA Activity in Plant Cells

**DOI:** 10.3389/fpls.2020.00816

**Published:** 2020-06-12

**Authors:** Michael Bennett, Jill Deikman, Bill Hendrix, Alberto Iandolino

**Affiliations:** Bayer AG, Woodland, CA, United States

**Keywords:** RNAi, dsRNA, barriers in plants, systemic silencing, local silencing

## Abstract

Foliar application of dsRNA to elicit an RNA interference (RNAi) response is currently under consideration as a crop protection strategy. To access the RNAi machinery of a plant, foliarly applied dsRNAs must traverse the plant cuticle, avoid nuclease degradation, and penetrate the cell wall and plasma membrane. Application methods and co-formulants have been identified by Bayer Crop Science researchers and others that can help bypass barriers to dsRNA uptake in plants leading to an RNAi response in greenhouse grown, young plants and cell cultures. However, these advances in dsRNA delivery have yet to yield systemic RNAi silencing of an endogenous gene target required for product concepts such as weed control. Systemic RNAi silencing in plants has only been observed with the *GFP* transgene in *Nicotiana benthamiana*. Because biologically meaningful whole plant RNAi has not been observed for endogenous gene products in *N. benthamiana* or in other plant species tested, under growing conditions including field production, the regulatory risk assessment of foliarly applied dsRNA-based products should not consider exposure scenarios that include systemic response to small RNAs in treated plants.

## Introduction

Weed control in crops using herbicides is one of the most important agronomic practices in modern agriculture. In order to work toward the development of biologically based herbicides, foliar application of double-stranded RNA (dsRNA) to plants was evaluated as a method to control weeds (Sammons et al., 2011) as well as plant pests and pathogens though a mechanism known as RNA interference (RNAi). To achieve the desired RNAi response and subsequent phenotype for weed control (including those with an herbicide-resistant phenotype), the foliarly applied dsRNA must ultimately be delivered into a sufficient number of responsive cells in the target organism. This dsRNA must travel from the surface of a leaf through the waxy cuticle, and then traverse the apoplast, cell wall, and plasma membrane to gain access to the plant cell’s RNAi machinery. Once inside the cell, the applied dsRNA can move to adjacent cells through plasmodesmata and subsequently to distal cells through the phloem vasculature. Alternatively, small dsRNAs (aka secondary or transitive siRNAs) generated through the cell’s RNAi machinery can also travel symplastically to the phloem vasculature and to distal cells. Distal movement of applied or generated dsRNA can result in distal or systemic RNAi thus facilitating efforts to employ dsRNA-based products to manage weeds. This differs from the proposed use of dsRNA as an agriculture insecticide where access to the plant cell’s RNAi machinery isn’t required to get the desired phenotype (Vogel et al., 2019). This document will summarize the current understanding of the barriers to efficient cellular delivery of nucleic acids (dsRNA and dsDNA) after foliar application to plants and some of the efforts to identify formulations that overcome these barriers. Specifically, this document will focus on the cuticle, nucleases, and cellular uptake as barriers, and will also discuss the requirements of the dsRNA structure to complete successful RNAi.

## Barriers to Efficient Foliar Uptake of dsRNA

### Cuticle as a Barrier

The plant cuticle is a lipophilic film generated by and covering the epidermis of leaves, young shoots, and fruit. The primary purpose of the cuticle is to prevent dehydration of plant surfaces. The cuticle is also known to impede the absorption of exogenous water and solutes (Schreiber, 2005). We hypothesize that it is these properties of the cuticle that make it resistant to dsRNA absorption, given the water solubility of dsRNA. To test this hypothesis, a fluorescently labeled (Cy3) 21 base pair (bp) siRNA was applied to the adaxial surface of Palmer Amaranth (*Amaranthus palmeri*) with 0.5% of the spreading surfactant Silwet L-77. Treated leaf cross-sections were collected at 4 h post application and assessed for the presence of Cy3-siRNA using fluorescence microscopy. A representative image obtained from this study is provided in the Supplementary Figure S1. Note that most of the applied Cy3-siRNA was found on the surface of the leaf. The fact that the Cy3-siRNA was mostly on the leaf’s surface was not surprising due to its size (MW > 14,000 Da for a 21-mer) and that it is a relatively water-soluble molecule.

**FIGURE 1:**
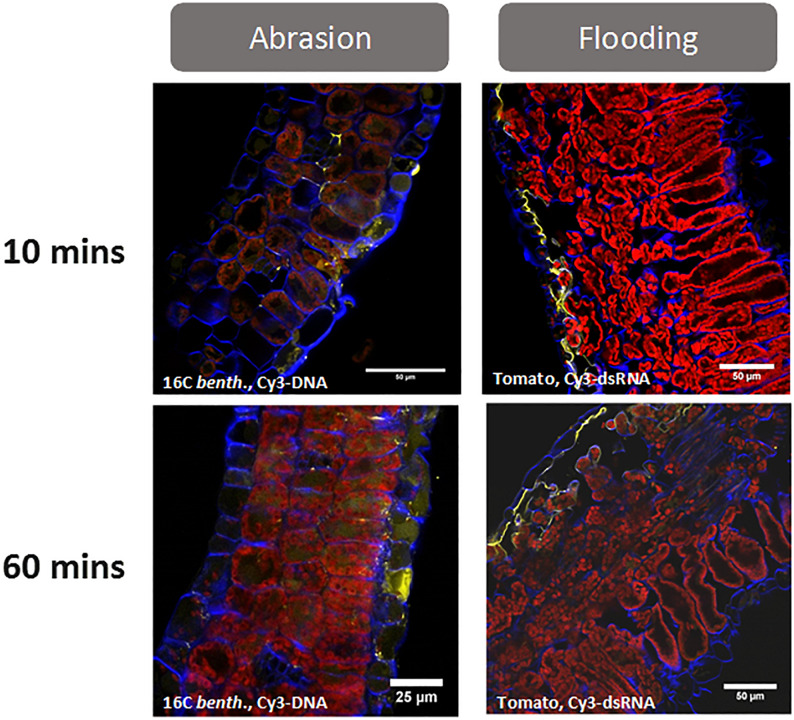
Uptake of fluorescently labeled (Cy3) nucleic acids (dsDNA-1 and siRNA-1 are GFP sequences, see Supplementary Table S1 for sequence information) after foliar application with either abrasion (*N. benthamiana*) or abaxial stomatal flooding (tomato). Leaf cross sections were collected at 10- and 60-min post application and visualized by confocal microscopy. The images show that both abrasion and abaxial stomatal flooding facilitate transcuticular movement of the applied nucleic acids within 10 min of treatment. Most of the Cy 3-labeled nucleic acids (yellow colored) can be found at the cells adjacent to the site of application and in the apoplast. While the difference between Cy3 and Cy3-labeled nucleic acid cannot be determined using a traditional confocal microscope, hyperspectral confocal fluorescence microscopy (Pedroso et al., 2009) was used in previous validation studies to confirm the yellow signal was due to Cy3 -labeled nucleic acid (unpublished Bayer Crop Science research). Abrasion was conducted using 0.5 mg.ml^–1^ Cy3-dsDNA^–1^ in water, 10 μl.leaf^–1^ and abraded with a 600-grit sandpaper. The stomatal flooding method is described in Supplementary Figure S2. For microscopy, a 5 mm punch from treated leaves was infiltrated in 4% paraformaldehyde/l×PBS followed by sucrose equilibration (10, 20, and 30% in l×PBS for 2–3 h each). Blue fluorescence represents cell wall, and red fluorescence is from chlorophyll.

To enhance cuticle penetration of siRNA and achieve robust visual RNAi phenotypes in this and other research (Dalakouras et al., 2016; Huang et al., 2018), abrasion, high pressure spraying, and abaxial stomatal flooding have been utilized. We have observed that spraying particles (celite, alumina, etc.) of sizes >2 microns with or after siRNA application at pressures <700 kPa resulted in improved cuticle penetration of the siRNA (Figure 1) and resulted in robust visual RNAi phenotypes (Huang et al., 2018). In addition to sprayed particles, other abrasive methods, such the use of sandpaper, resulted in improved siRNA penetration and RNAi silencing after adaxial foliar siRNA application. Consistent with these results, Dalakouras et al. (2016) did not observe RNAi silencing of green fluorescent protein (GFP) phenotypes in *Nicotiana benthamiana* in the absence of tissue wounding. They accomplished this either biolistically with gold particles (1 μm) and a gene gun or by spraying an aqueous siRNA solution at high pressure (7–8 bar) and close (2–4 cm) to the plant surface. Dalakouras et al. (2016) reported that they did not observe GFP silencing by either pipetting the solution to the adaxial surface of the plant or by infiltration. This lack of dsRNA delivery with infiltration suggests that abrasion and high-powered spray must be impacting other aspects of siRNA delivery in addition to cuticle penetration.

Successful siRNA delivery resulting in robust visual RNAi endogenous gene and transgene phenotypes in several plant species has been demonstrated using either pipet application (Supplementary Figure S2) or relatively low pressure (70–140 kPa) spray (not shown) to the abaxial leaf surface. This was accomplished by including >0.3% of a super spreading surfactant, such as Silwet L-77^1^, in the siRNA solution (0.1–1.0 mg/mL) at the time of application, which drives uptake of the dsRNA by stomatal flooding. An example of stomatal flooding siRNA delivery and resulting GFP silencing are featured in Supplementary Figure S2. It should be noted that the application conditions, e.g., application to the abaxial surface and surfactant concentrations, utilized in these experiments are not employed in commercial agriculture.

### Nuclease Stability as a Barrier

*In vivo* nuclease degradation is known to impact the efficiency of applied siRNA to elicit an RNAi response in mammalian systems (Behlke, 2008). Not much is known about the impact of nucleases on the delivery efficiency of foliarly applied siRNAs in plants; however, the well documented presence of nucleases in plants suggests that nuclease degradation could impact the ability of a foliarly applied siRNA to gain access to the plant cell (Pérez-Amador et al., 2000). To investigate this, we applied 22 bp siRNA by syringe infiltration to an expanded leaf of *N. benthamiana*. Tissue samples from the infiltration site were collected at 0, 1, 2, 4, 6, and 8 h post application. Analysis of RNA extracts obtained from the collected tissues by anion exchange HPLC revealed that applied siRNA was not detected at 6 h post application (Supplementary Figure S3).

**FIGURE 2:**
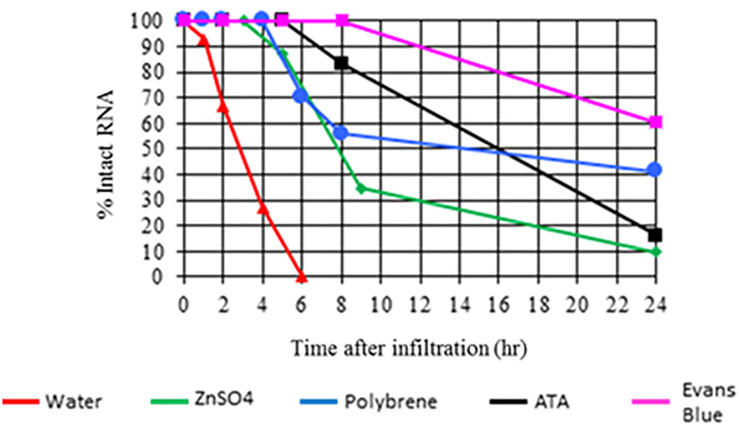
Impact of nuclease inhibitors and polybrene on *in planta* persistence of applied 22 bp siRNA (siRNA-2 from GFP, see Supplementary Table S1 for sequence information and additional experiment information). Aqueous solutions of siRNA-2 either alone or with a nuclease inhibitor or polybrene were applied to expanded leaves of *N. benthamiana* by syringe infiltration. Tissue samples were collected from the infiltration site at 0, 1, 2, 4, 6, 8, and 24 h after application. The applied siRNA was extracted from the samples and analyzed by anionic exchange HPLC. Results indicate that siRNA applied without a nuclease inhibitor or polybrene was completely degraded by 6 h. siRNA applied with nuclease inhibitors or polybrene was still detectable at 24 h.

In a similar experiment, 22 bp siRNA was infiltrated in the presence or absence of a nuclease inhibitor or the cationic polymer polybrene. Tissue samples were collected from the infiltration site at 0, 1, 2, 4, 6, 8, and 24 h post application and analyzed using anionic exchange HPLC. As with the experiment mentioned above, siRNA applied in the absence of a nuclease inhibitor or polybrene was not detectable at 6 h post application (Figure 2). siRNA applied with a nuclease inhibitor or polybrene which binds dsRNA was still detectable at 24 h. Both sets of experimental results strongly suggest that nucleases act as a barrier to efficient dsRNA delivery. This assertion is further supported by recent publications demonstrating that agents such as carbon dots, single-walled carbon nanotubes, and clay nanosheets enhance nuclease stability and delivery efficiency of applied nucleic acids in plants (Mitter et al., 2017; Demirer et al., 2019; Schwartz et al., 2019).

### Cellular Uptake as a Barrier

Plant cells have both a cell wall and plasma membrane that can pose as barriers to efficient nucleic acid delivery. The plant cell wall is a matrix comprised of cellulose, hemicelluloses, pectin, and other biopolymers. It provides support and protection, and acts as a filtering mechanism for plant cells. The cell wall pore size is primarily dependent on environmental factors, plant species, and cell type (Carpita et al., 1979). Macromolecules such as globular proteins (MW≈17 kDa) and extracellular polysaccharides (MW > 100 kDa) have been shown to pass through plant cell walls (Bauer et al., 1973; Carpita et al., 1979). To characterize the nucleic acid exclusion limit for plant cells, we incubated GFP-expressing BY-2 suspension cells with and without flg22 (a 22-amino acid flagellin fragment) and either a 21, 50, or 90 bp (MW = 12.9 kDa, 32.8 kDa, and 55.5 kDa, respectively) fluorescently labeled (pHRodo) double-stranded DNA (dsDNA). DNA was used as a surrogate for double-stranded RNA in this study because of challenges getting synthesized RNAs >48 bp from commercial sources. DNA has similar physiochemical attributes (charge density, hydrophilicity, etc.) as RNA that make it a suitable replacement in delivery studies. Flg22 is known to stimulate ligand-induced endocytosis in plants. Uptake of the applied DNA by formed endosomes would be an indication that it was able to successfully pass through the cell wall thus gaining access to the plasma membrane. Fluorescent imaging of the treated BY-2 cells indicated that the 21 and 50 bp DNAs were more readily taken up by flg22-stimulated endosomes than the 90 bp DNA (Supplementary Figure S4). This result indicates an exclusion limit of between 32.8 and 55.5 kDa for nucleic acids in BY-2 cells. It should be noted that none of the DNAs screened were internalized by the cells without flg22 stimulation, suggesting that the plasma membrane is also a barrier to efficient cellular uptake and that transfection agents are required for nucleic acid delivery to plant cells. Transfection agents have been shown to improve both DNA and RNA delivery efficiency in plant cells (Bennett et al., 2017; Demirer et al., 2019; Schwartz et al., 2019).

To achieve the robust and reproducible whole plant RNAi phenotypes necessary for weed and plant virus control product concepts, the applied dsRNA or the resultant secondary/transitive sRNAs must be able to assert their activity throughout the plant, including tissues not accessible to topical application. Identification of co-formulants that enable foliar applied dsRNA to overcome the barriers listed above have indeed yielded improvements in both dsRNA delivery efficiency and RNAi triggered endogenous gene and transgene phenotypes in plants (Huang et al., 2018). Literature examples of robust and reproducible whole plant RNAi after foliar application of dsRNA have been limited to transgene silencing in plant species such as *N. benthamiana* (Supplementary Figure S5; Dalakouras et al., 2016; Huang et al., 2018). Other examples in the literature of whole plant endogenous gene silencing phenotypes through foliar application of exogenous nucleic acid application involved the use of biological vectors such as viral induced gene silencing (VIGS) (Burch-Smith et al., 2006). Application of such vectors would likely not be suitable for commercial applications, due to the additional regulatory burden and region-specific acceptance of crops with biotechnology derived traits.

We have made progress toward achieving robust and reproducible gene silencing in treated leaves of plants by identifying methods and co-formulants that overcome the barriers to efficient delivery of foliarly applied dsRNA delivery. However, these methods, which result in both local (where applied to leaf) and systemic (untreated leaf) silencing of a GFP transgene in 16C *N*. *benthamiana*, result only in local silencing of the GFP transgene in other plant species such as *Arabidopsis thaliana* and for endogenous genes such as magnesium chelatase in *Amaranthus cruentus* (Supplementary Figure S5). Attempts by others to improve the movement of applied dsRNA through the plant’s vasculature and achieve systemic distribution and RNAi in tissues not readily accessible to a spray such as the apical meristem have not been successful (Dalakouras et al., 2018). In research published by Dalakouras et al. (2018) exogenous dsRNA was applied directly to the vasculature by petiole absorption of 16C *N*. *benthamiana* and other plants. This approach was successful in achieving systemic distribution of exogenous dsRNA, however, it did not result in down regulating the transgene product levels since the observed movement was through the apoplast and xylem. Cellular uptake and movement through the phloem was not observed so the applied dsRNA did not have access to the dicer-like endonucleases or other RNAi pathway enzymes required for initiating RNAi (Dalakouras et al., 2018). Distribution such as this through the apoplast and xylem might be applicable for pest management by targeting RNAi in insects and fungi (Koch et al., 2016; San Miguel and Scott, 2016). It is not useful, however, to achieve the whole plant silencing phenotypes desired for weed and plant virus control product concepts.

### dsRNA Properties Constrain RNAi Activity in Planta

In addition to containing sequences that match the target gene, the specific dsRNA size and structure is important for successful local or systemic down-regulation of a plant gene product from topically applied dsRNA.

Down-regulation of targeted genes using topically applied RNAs has been observed with dsRNAs from 21 to ∼150 bp, but 22 bp siRNAs produced the strongest silencing phenotypes (Dalakouras et al., 2018; Hendrix et al., 2020; unpublished Bayer Crop Science data). It is hypothesized that 22 bp siRNAs provide the greatest silencing efficiency, compared to the other dsRNAs tested, because they can induce production of secondary (transitive) siRNAs for the targeted mRNAs, which could increase the number of active silencing siRNAs for that gene target (Chen et al., 2010; Cuperus et al., 2010). Topically applied 22 bp siRNAs induced production of secondary siRNAs homologous to the target mRNA for all targeted genes examined, although the amount of secondary siRNAs varied by gene (Hendrix et al., 2020). These 22 bp siRNAs also generated a greater silencing phenotype in the leaf to which they were applied compared to 21 bp siRNAs for a variety of targeted genes. Importantly, 22 bp siRNAs, but not 21 bp siRNAs, were also able to trigger systemic silencing for the GFP transgene in *N. benthamiana* (Dalakouras et al., 2016; Hendrix et al., 2020), which is discussed further below.

In our research we observed that 2 bp 3′ overhangs are required for strong silencing activity of topically applied small to mid-sized dsRNAs (21 – 60 bp) in plant cells (unpublished Bayer Crop Science research). These overhangs are particularly sensitive to degradation by RNAses found in plant tissues (unpublished Bayer Crop Science research), and so these structures may not be very long-lived in the environment.

Work in Bayer laboratories has shown that not every target gene can be successfully down-regulated using topically applied siRNAs or dsRNAs. Reduced mRNA concentration was observed for only 40% of gene products targeted, after testing at least 4 homologous 22 bp siRNAs per gene (unpublished Bayer Crop Science research). A weak correlation between target gene expression level and ability to down-regulate that gene with topically applied siRNA suggested that highly expressed genes may be more readily silenced. However, reliable predictors of targets that can be silenced using topical RNA have not yet been identified. Other hypotheses for the limitation on silencing of targets is that secondary structure of the mRNA *in vivo* (Ding et al., 2013), or protein interactions with an mRNA may limit accessibility of the sequence to a dsRNA (Liu et al., 2014).

Further, not all 22 bp siRNAs against responsive targets are efficacious. Rules for efficacious siRNAs have been described in the literature based on research with mammalian cells (Reynolds et al., 2004; Jagla et al., 2005), and some of these are expected to be important for plant cells also. However, only approximately half of siRNAs that were selected based on such guidelines were efficacious in reducing mRNA concentration for a targeted gene that could be silenced (unpublished Bayer Crop Science research). More recently, an *in vitro* assay was developed to identify efficacious siRNAs (esiRNAs) targeting a plant virus gene (Gago-Zachert et al., 2019). The two critical features for the esiRNA were ability to bind Argonaute proteins and the ability to access the target RNA. The *in vitro* assay correlated well with *in planta* anti-viral activity. However, further refinement of rules for selecting *in silico* which siRNAs will be efficacious for silencing genes would be useful.

### Systemic Silencing Using Topical dsRNA Has Only Been Documented for the GFP Transgene in *N. benthamiana*

Systemic RNAi has been observed for the GFP transgene in *N. benthamiana* line 16C (Ruiz et al., 1998) using topical RNA with dsRNAs that are 124 bp or 22 bp, but not 21 bp (Dalakouras et al., 2016; Schwartz et al., 2019; Hendrix et al., 2020; unpublished Bayer Crop Science research). The ability of 22 bp siRNAs to cause systemic silencing is likely related to their ability to induce production of secondary sRNAs (Chen et al., 2010; Cuperus et al., 2010; Dalakouras et al., 2020; Hendrix et al., 2020). Activity by longer dsRNAs to induce systemic silencing may result from generation of 22 bp siRNAs by dicer-like enzymes in plant cells.

Strongly expressed GFP transgenes have been targeted in tomato and *Arabidopsis* by topical application of 22 bp siRNAs, and while strong local silencing was achieved, no systemic silencing was ever observed those species (Supplementary Figure S5 and unpublished Bayer Crop Science research).

A number of endogenous genes were down-regulated with topically applied siRNAs in *N. benthamiana*, but systemic silencing was not observed (unpublished Bayer Crop Science research). Endogenous genes that have been targeted in *N. benthamiana* included magnesium cheletase subunit H (CHL-H), magnesium cheletase subunit I, GUN4, and phosphoribosylanthranilate transferase (Hendrix et al., 2020; and unpublished Bayer Crop Science research). Visible local silencing and/or reduced mRNA levels were observed after targeting each of these gene products in *N. benthamiana*, but no systemic phenotypes were detected. Endogenous genes including CHL-H, HSP70, Ubiquitin B and others were down-regulated in Amaranth species and strong local phenotypes were evident, but no systemic RNAi response was observed (Supplementary Figure S5 and unpublished Bayer Crop Science research). In Supplementary Figure S5C, magnesium chelatase silencing can be observed as yellow spots in several leaves after treatment of young plants with *CHL-H* siRNA delivered with the particle spray method. All of the leaves showing silencing of magnesium chelatase were present at the time of treatment, and all observed silencing phenotypes can be explained by direct delivery of siRNA. In canola, the strongly expressed CP4 gene that conveys tolerance to the herbicide glyphosate was silenced locally but no systemic silencing was observed (unpublished Bayer Crop Science research).

The presence of introns in most endogenous genes may limit their susceptibility to systemic gene silencing (Christie et al., 2011; Dadami et al., 2014; Dalakouras et al., 2020). However, McHale et al. (2013) showed systemic silencing of the endogenous chalcone synthase gene in *Arabidopsis* using a 22-nt artificial miRNA. Further work should be done to better understand the role of introns in systemic gene silencing.

Currently, the underlying reason(s) for the limitation of systemic RNAi response by topical dsRNA application to only the GFP transgene and only in 16C *N. benthamiana* is unclear.

## Conclusion

Plant tissues have many barriers to entry of foreign and topically applied nucleic acids. Even if a sprayed dsRNA is formulated or applied in a manner that helps to bypass the plant’s physical (e.g., cuticle, cell wall) and biochemical barriers (e.g., nucleases) and does enter a plant cell, many molecular barriers exist that must be overcome to generate a biologically meaningful RNAi response from the plant. As with all RNAi, the first requirement is that the nucleic acid sequence is complementary to the sequence of a gene product that is expressed in the cell that received the dsRNA; however, our research demonstrated that sequence match alone does not guarantee a biologically meaningful RNAi response. The structure of the dsRNA needs to be efficacious for an RNAi response, including presence of 2 nt 3′ overhangs - which tend to be degraded by plant or microbial nucleases. Finally, even if local down-regulation of a gene product occurs, evidence from this research as well as the literature suggests that a systemic RNAi response would be very unlikely to occur. Therefore because biologically meaningful whole plant RNAi has not been observed for endogenous gene products in *N. benthamiana* or in other plant species tested, under growing conditions including field testing, the regulatory risk assessment of foliarly applied dsRNA-based products should not consider exposure scenarios that include systemic response to small RNAs in treated plants.

## Data Availability Statement

The datasets generated for this study will not be made publicly available, the authors are willing to accept requests for data sets referenced in this article. However, since legal intellectual property review may be necessary to release the data sets, the authors cannot commit to release the data sets at this time.

## Author Contributions

MB and JD contributed equally to the research reported and wrote the manuscript. BH and AI contributed to the plant uptake and siRNA testing work reported in this manuscript.

## Conflict of Interest

The research reported was funded by Bayer Crop Science and the researchers involved in this work were employees of Bayer Crop Science and its predecessors in business.

## Footnotes

1 Note this level of Silwet L-77 is at least a level of magnitude higher than normally used in agriculture.
