# Supplementary material for: Barriers to Efficient Foliar Uptake of dsRNA and Molecular Barriers to dsRNA Activity in Plant Cells
Source: Front Plant Sci. 2020 Jun 12;11:816. doi: 10.3389/fpls.2020.00816 (PMC7304407; doi:10.3389/fpls.2020.00816)
Supplement: Supplementary file 1 [file Table_1.DOCX]

**A B**


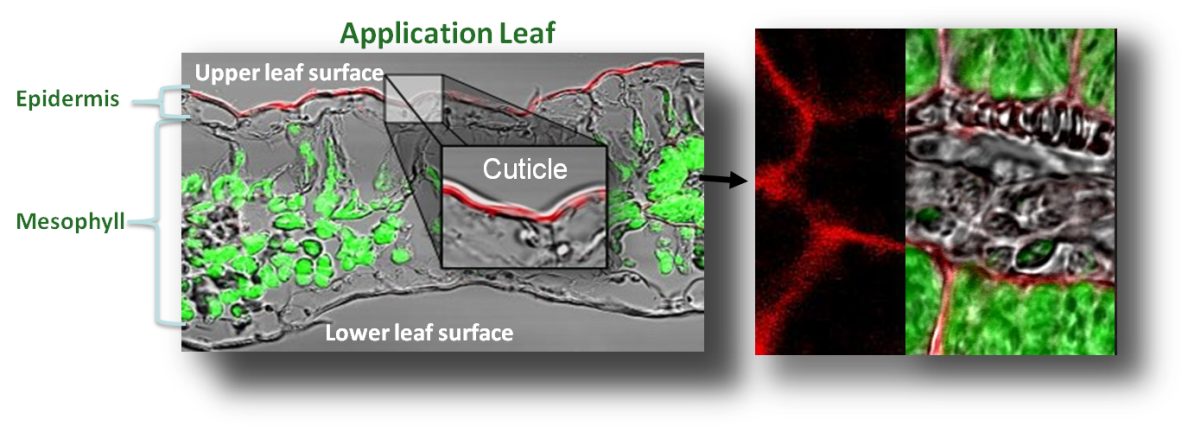


RNA =

**Figure S1**. Uptake of fluorescently-labeled (Cy3)-siRNA-3 (21 bp, Ap.EPSPS sequence, see Table 1 for more information) after application with 0.5% Silwet L-77 to the adaxial surface of Palmer Amaranth. (A) A majority of the siRNA-1 (Red) was associated with the cuticle 4 hours post application. (B) siRNA-3 that penetrated the cuticle was primarily found in the apoplast.

**60 mins**

**3-5 days**

**C**

**B**

**A**

**GFP gene silencing**

**5 days after application**

**Figure S2**. Delivery of siRNA-1 targeting the GFP transgene (Supplementary Table 1) to transgenic GFP-expressing tomato by abaxial stomatal flooding resulted in RNAi silencing phenotypes. (A) An siRNA solution containing > 0.3% Silwet or other superwetting surfactant and a sugar, salt, or other osmolyte is applied by either hand pipette or spray to the abaxial leaf surface (Formulation example: 1 mg.ml^-1^ siRNA-1, 200 mM sucrose, 4mM MES buffer at pH 5.7, and 0.3% Silwet L77 surfactant). (B) The surfactant drives uptake of the dsRNA through the stomata and into the leaf where it has access to the cells of interest resulting in (C) the desired phenotype (GFP silencing as pictured) at 3-5 days post application.

Red: dsRNA Standard

Blue: 0 hrs (100 % as control)

Green: 1 hrs ( 95.9 %)

Pink: 2 hrs (57.0 %)

Black: 4 hrs (29.3 %)

Light blue: 6 hrs (Not detectable)

Orange: 8 hrs (Not detectable)

Sea blue: Negative control

Applied dsRNA

**Figure S3.** Stability of applied 22 bp siRNA (siRNA-2, see Supp. Mat. Table 1 for sequence information and additional experiment information) in *N. benthamiana.* siRNA-2 was applied by syringe infiltration. Tissues samples were collected at 0, 1, 2, 4, 6, and 8 hours post application. Extracted dsRNA was analyzed by anionic exchange HPLC and showed evidence of nuclease degradation over time. The applied dsRNA was undetectable in tissues collected at 6 hours post application.


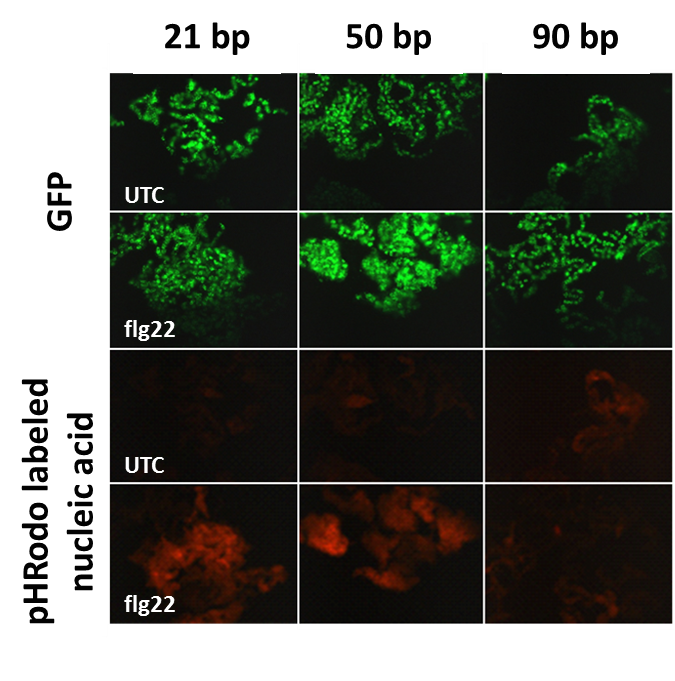


**Figure S4**. Cellular uptake is dependent on nucleic acid size. GFP-expressing BY-2 suspension cell cultures were incubated with flg22 (a 22-amino acid flagellin fragment) and either 21 (dsDNA-2), 50 (ds-DNA-3), or 90 (dsDNA-4) bp blunt ended, chemically synthesized, dsDNA from the *Amaranthus palmeri* EPSPS gene (see Supplementary Table 1 for sequence information and additional experiment information). UTC is an untreated control. Flg22 is known to stimulate ligand induced endocytosis in plants. Uptake of the fluorescently labeled nucleic acid by flg22-stimulated cells is an indication of applied nucleic acid penetration through the cell wall. The bottom row of images in the above figure show greater pHRodo (red) fluorescence in cells treated with a labeled 21 or 50 bp dsDNA than with the 90 bp dsDNA suggesting that the size exclusion limit for BY2 cell walls is between 50 – 90 bp.

**Figure S5.** Examples of silencing of a transgene (GFP) in 16C *N. benthamiana* (A, siRNA-2) and *A.* *thaliana* (B, siRNA-2), and an endogenous gene (magnesium chelatase H) in *A. cruentus* (C, siRNA-4) achieved using abrasion-based delivery methods (Huang *et al*, 2018; see Table 1 for sequence information). GFP silencing in 16C *N. benthamiana* resulted in both local (*) and systemic (**) phenotypes in treated and untreated leaves, respectively. Phenotypes have only been observed in application leaves when a GFP transgene was targeted in *A.* *thaliana* or when an endogenous gene such as magnesium chelatase H was targeted in *A. cruentus* (*). Silencing phenotypes in *A. cruentus* leaves were determined to be from primary delivery of siRNAs targeting *CHL-H*, as silencing only occurred in leaves formed and accessible to the spray at the time of the particle-spray treatment. siRNA-2 was delivered by abrading leaves with a 660-grit sandpaper rolled on leaves after application of the siRNA with a 0.05% Silwet L77 solution to facilitate surface spreading (Huang *et al*, 2018). siRNA-4 was delivered to *A. cruentus* seedlings (C) with a particle slurry sprayed onto developing leaves with a hand held airbrush sprayer (Master Airbrush G23) at an air pressure of 25 PSI . The following formulation composition was used: 4 mg.ml^-1^ siRNA-4, 50 mM Na oxalate, 10 mg.ml^-1^ Celite^®^503 and 0.1% xantham gum.

*

*

*

*

**Table 1:** Nucleic acids used in the reported experiments:

Note: All Cy3-labelled nucleic acids were chemically synthesized and labeled by IDT (Integrated DNA Technologies, Inc., Coralville, IA, USA). An in-house Bayer Crop Science trade secret method was used to synthesize other nucleic acids. b-b = blunt ended, o-o = 2-bp overhangs at both ends. Av = *Aequorea victoria*, Ap = *Amaranthus palmeri*, Ac = *Amaranthus cruentus*.

**Additional information on delivery methods**

**Syringe infiltration of N. benthamiana leaves (Figure 2 and S3).**

100 μl of a 1 μM nucleic acid solution containing 10 mM MES and 10 mM MgCl_2_ was syringe infiltrated into a discrete region of a leaf clearly marked as the infiltrated area. A 4 mm diameter leaf punch was collected from each infiltrated leaf at pre-determined intervals. The total leaf RNA was extracted with 1 ml Trizol following manufacturer instructions. The presence of the intact nucleic acid was analyzed using anion exchange HPLC.

**Labeled nucleic acid uptake via flg22 mediated endocytosis in BY-2 suspension cells**

An equal volume of a ROS100 medium (34.2 mg.ml^-1^ sucrose, 5.9 mg.ml^-1^ HEPES and 0.1 M CaCl_2_ adjusted to pH 7.4) was added to an aliquot of 4-6 day old BY-2 suspension cell culture and centrifuged 2 min at 100X g. Then the supernatant was removed and washed with 2X original volume of ROS100 medium and centrifuged as above. Cells were then resuspended in the original volume of ROS100 medium and a 100 μl aliquot was added per well in a non-binding 96-well flat, clear bottomed plate (Corning 3651) and incubated for 1 hr in the dark at 26^o^C to equilibrate the cells. Next, Cy3-labeled nucleic acids (dsDNA-2/3/4) or Cy3-labeled nucleic acid + flg22 were added according to the respective experimental treatments and mixed by pipetting with a wide-bore pipette tip. Plates were then covered with optical tape (Life tech. cat no. 4311971) and placed in a plate reading fluorometer (SpectraMax M5). Light absorbance at 600 nm was measured to assess initial GFP fluorescence. The fluorometer was then set at EX/EM 560/585 to read pHRodo fluorescence from the bottom of the plate every 10 min overnight (~16hours) at 26^o^C. At the end of the overnight experiment, the medium was removed and 100 μl of fresh ROS100 medium was added together with 2.5 μl of 0.4% trypan blue solution (final concentration is 0.01%) to determine cell viability. Finally, end point pHRodo fluorescence (EX/EM 560/585) readings were taken from the bottom of the plate and cells were visualized under a microscope.
